# NLRP3, NLRP12, and IFI16 Inflammasomes Induction and Caspase-1 Activation Triggered by Virulent HSV-1 Strains Are Associated With Severe Corneal Inflammatory Herpetic Disease

**DOI:** 10.3389/fimmu.2019.01631

**Published:** 2019-07-16

**Authors:** Pierre-Gregoire Coulon, Nisha Dhanushkodi, Swayam Prakash, Ruchi Srivastava, Soumyabrata Roy, Nuha I. Alomari, Angela M. Nguyen, Wasay R. Warsi, Caitlin Ye, Edgar A. Carlos-Cruz, Uyen T. Mai, Audrey C. Cruel, Keysi M. Ekmekciyan, Eric Pearlman, Lbachir BenMohamed

**Affiliations:** ^1^Laboratory of Cellular and Molecular Immunology, School of Medicine, Gavin Herbert Eye Institute, University of California, Irvine, Irvine, CA, United States; ^2^School of Medicine, Institute for Immunology, University of California, Irvine, Irvine, CA, United States; ^3^Department of Molecular Biology and Biochemistry, School of Medicine, University of California, Irvine, Irvine, CA, United States

**Keywords:** inflammasome, NLRP3, NLRP12, IFI16, HSV-1, IL-1β, herpetic keratitis and cornea

## Abstract

The crosstalk between the host's inflammasome system and the invading virulent/less-virulent viruses determines the outcome of the ensuing inflammatory response. An appropriate activation of inflammasomes triggers antiviral inflammatory responses that clear the virus and heal the inflamed tissue. However, an aberrant activation of inflammasomes can result in a harmful and overwhelming inflammation that could damage the infected tissue. The underlying host's immune mechanisms and the viral virulent factors that impact severe clinical inflammatory disease remain to be fully elucidated. In this study, we used herpes simplex virus type 1 (HSV-1), the causative agent of corneal inflammatory herpetic disease, as a model pathogen to determine: (i) Whether and how the virulence of a virus affects the type and the activation level of the inflammasomes; and (ii) How triggering specific inflammasomes translates into protective or damaging inflammatory response. We showed that, in contrast to the less-virulent HSV-1 strains (RE, F, KOS, and KOS63), corneal infection of B6 mice with the virulent HSV-1 strains (McKrae, 17 or KOS79): (i) Induced simultaneous expression of the NLRP3, NLRP12, and IFI16 inflammasomes; (ii) Increased production of the biologically active Caspase-1 and pro-inflammatory cytokines IL-1β and IL-18; (iii) Heightened recruitment into the inflamed cornea of CD45^high^Ly6C^+^Ly6G^−^F4/80^+^CD11b^+^CD11c^−^ inflammatory monocytes and CD45^high^CD11b^+^F4/80^−^Ly6G^hi^Ly6C^med^ neutrophils; and (iv) This intensified inflammatory response was associated with a severe corneal herpetic disease, irrespective of the level of virus replication in the cornea. Similarly, *in vitro* infection of human corneal epithelial cells and human monocytic THP-1 cells with the virulent HSV-1 strains triggered a synchronized early expression of NLRP3, NLRP12 and IFI16, 2 h post-infection, associated with formation of single and dense specks of the adapter molecule ASC in HSV^(+)^ cells, but not in the neighboring bystander HSV^(−)^ cells. This was associated with increased cleavages of Caspase-1, IL-1β, and IL-18. These findings suggest a previously unappreciated role of viral virulence in a synchronized early induction of the NLRP3, NLRP12, and IFI16 inflammasomes that lead to a damaging inflammatory response. A potential role of common virus virulent factors that stimulate this harmful inflammatory corneal disease is currently under investigation.

## Introduction

Inflammasomes are multiprotein complexes that form in the cytosol of the host cells following sensing of invading infectious pathogens (1). Activation of the inflammasomes system following an early encounter with viral pathogens can mediate a protective or harmful inflammatory response [reviewed in Kanneganti (2)]. The five major canonical inflammasomes (NLRP3, NLRP6, NLRP12, IFI16, and AIM2) include cytoplasmic and nuclear sensor molecules that form a complex with the adaptor protein ASC (apoptosis-associated speck-like protein containing CARD), and the effector protein pro-caspase-1 [reviewed in Kanneganti (2)]. Early, in response to a viral infection, appropriate activation of one or several inflammasomes induces the maturation and secretion of biologically active pro-inflammatory interleukins IL-1α, IL-1β, IL-18, and IL-33, which in turn activate inflammatory cell recruitment to the site of viral infection (3, 4). An optimal activation of inflammasomes can induce a sub-clinical inflammatory response that is beneficial to the well-being of the cornea. However, a dysregulation or a hyper-activation of inflammasomes can lead to clinical inflammation that exacerbates corneal diseases (5–7). The underlying cellular and molecular inflammatory mechanisms and the viral genetics that impact clinical inflammation and the severity of herpetic disease remain to be fully elucidated.

In the present study, we used herpes simplex virus type 1 (HSV-1) infection, the causative agent of cornea inflammatory herpetic disease, as a model pathogen, to determine whether and how viral virulence affects activation of the inflammasome system that result in inflammatory disease. The HSV-1 infects a staggering 3.72 billion individuals worldwide (i.e., over 52% of the world's population) (8–10). HSV-1 infection of the cornea can lead to subclinical inflammation that develops into minor epithelial herpes keratitis and can easily be resolved by the innate and adaptive immune system (11, 12). However, the infection may spread deeper in corneal stroma and develop into a more severe inflammatory disease (i.e., clinical inflammation) (11, 12). If left untreated with antiviral eye drops (e.g., Acyclovir and derivatives) or with steroid eye drops, this clinical inflammation can cause blinding recurrent herpetic stromal keratitis (rHSK), a common indication for corneal transplantation (13).

We found that, unlike infection with the less-virulent strains of HSV-1 (i.e., RE, F, KOS, and KOS63), corneal infection of B6 mice with the virulent strains (i.e., McKrae, 17, and KOS79) that produced severe corneal inflammatory herpetic disease, was associated with high and early expression of NLRP3, NLRP12, and IFI16 inflammasomes. This was associated with increased production of biologically active Caspase-1 and pro-inflammatory cytokines IL-1β and IL-18 along with a heightened recruitment into the inflamed cornea of inflammatory CD45^high^Ly6C^+^Ly6G^−^F4/80^+^CD11b^+^CD11c^−^ monocytes and CD45^high^CD11b^+^F4/80^−^Ly6G^hi^ neutrophils (PMN). Moreover, unlike *in vitro* infection of the human corneal epithelial cell line (hTCEpi) with the less-virulent strains RE, F, KOS, and KOS63, the virulent strains McKrae, 17, and KOS79 led to: (i) High levels of activated NLRP3, NLRP12, and IFI16 inflammasomes; (ii) Increased formation of ASC specks; and (iii) High levels of IL-1β and IL-18. We propose that early during corneal infection with HSV-1, a crosstalk between the host's inflammasome system and virulent/less-virulent strains of HSV-1 determines the outcome of the ensuing inflammatory corneal herpetic disease (i.e., either clinical or subclinical inflammation). Virulent strains of HSV-1 may use one or several yet-to-be determined virulent factors to activate multiple inflammasomes and this translates into a harmful corneal inflammatory herpetic disease.

## Materials and Methods

### Mice

C57BL/6 (B6) wild type (WT) mice (6–8 weeks old) were purchased from The Jackson Laboratory (Bar Harbor, ME). Both male and female mice at 6–8 weeks were used for this study (with the same male/female ratio in each group). Animal studies were conducted with the approval of the Institutional Care and Use Committee of University of California-Irvine (Irvine, CA) and conformed to the Guide for the Care and Use of Laboratory Animals published by the US National Institute of Health (IACUC protocol #2002-2372).

### Cell Lines and Viruses

The cell line hTCEpi (hTERT immortalized human corneal epithelial cell line) (kind gift from Dr. James Jester, Department of Ophthalmology, University of California-Irvine) were grown in complete—supplemented—keratinocyte growth medium 2 (PromoCell, Cat. No. C-20211 supplemented with C39011). The human monocytic cell line THP-1 (14) were grown in RPMI-1640 (HyClone, GE Healthcare) supplemented with 10% FBS and 1% penicillin-streptomycin, plus Sodium Pyruvate 1mM (Lonza, Switzerland) and L-Glutamine 2mM (Corning, Manassas, VA). They were then differentiated into macrophages following a 2 days stimulation with 25 ng/ml PMA (Sigma-Aldrich, St. Louis, MO), followed by subsequent resting in fresh medium without PMA for additional 3 days. Rabbit skin (RS) cells (ATCC, VA) were grown in Minimum Essential Medium Eagle with Earl's salts and L-Glutamine (Corning, Manassas, VA – Cat. No. 25-025_CI) supplemented with 5% fetal bovine serum and 1% penicillin-streptomycin. HSV-1 “high-passage laboratory strains” McKrae, Strain17, KOS, RE, F and “low-passage clinical isolates” KOS79 and KOS63 (generously gifted by Dr. Richard D. Dix, Department of Biology, Georgia State University, Atlanta, GA) were propagated in RS cells as described previously (15). All the virus strains were purified by ultracentrifugation in sucrose gradient and titrated by plaque assay in RS cells. All seven strains of HSV-1 were used for the *in vitro* and *in vivo* experiments.

### *In vitro* HSV-1 Infection of Cultured Cells

hTCEpi cells were grown to sub-confluent monolayers in 6-well plates (2 million cells/well). For THP-1 infection, the cells were first seeded in 6-well plates (2 million cells/well), treated with PMA for 2 days and infected 5 days later. Infections with various HSV-1 strains were performed at 1 MOI (for hTCEpi) or 10 MOI (for THP-1 cells) after 1 h incubation (in FBS-free media) at 37°C with intermittent rocking. The cells were then rinsed and overlaid with fresh medium. The cultures were terminated at various time points (2, 8, and 24 h post-infection) in replicates for FACS analysis and Immunoblotting. Culture supernatants and cells lysates (obtained using RIPA buffer supplemented with protease and phosphatase inhibitors—Thermo Scientific, Cat. No. 89900 and Cat. No. 78442) were stored at −80°C for ELISA and immunoblotting, respectively. IL-1β ELISAs (Quantikine kit, Cat. No. DLB50) were carried out as per manufacturer's instructions (R&D Systems, Inc., Minneapolis, MN).

### Ocular Infection With Virulent and Less-Virulent Strains of HSV-1

Mice were infected with 2 × 10^5^ pfu per eye of each virulent (McKrae, 17 or KOS79) and less-virulent (KOS, RE, F or KOS63) strain of HSV-1 *via* eye drops for a total of 3 μL sterile PBS after corneal scarification (i.e., light crosshatched pattern of 4–5 vertical and 4–5 horizontal scratches using a 25-gauge needle). Following ocular infection, mice were monitored daily for ocular herpes infection and disease progression.

### Monitoring of Ocular Herpes Infection and Disease in Mice

Virus shedding was quantified in eye swabs collected on days 2, 5, 7, 9, and 14. Eyes were swabbed using moist type 1 calcium alginate swabs and frozen at −80°C until titrated by plaque assay on RS cell monolayers. Briefly, RS cells were grown to 90% confluence in 24-well plates. Transfer medium (in which eye swabs were stored) was added after appropriate dilution at 300 μl per well in 24-well plates. Infected monolayers were incubated at 37°C for 1.5 h and were rocked every 15 min for viral adsorption and then overlaid with medium containing carboxy-methyl cellulose. After 48 h of incubation at 37°C, cells were fixed and stained with crystal violet, and viral plaques counted under a light microscope. Positive controls were run with every assay using previously tittered laboratory stocks of McKrae.

Animals were examined for signs of recurrent corneal herpetic disease (blepharitis score for eyelid inflammation and keratitis score for corneal cloudiness) by slit lamp and scored according to a standard 0–4 scale (0 = no disease; 1 = 25%; 2 = 50%; 3 = 75%; 4 = 100%) as previously described (16, 17). Pictures were taken at day 2, 5, 8, 10 and 14 post infection using a Nikon D7200 camera with an AF-S Micro NIKKOR 105 mm f/2.8 lens and a Wireless Remote Speedlight SB-R200 installed.

### Flow Cytometry and FLICA Staining on Corneal Cells Infiltrate

At day 2 and 8 post infection, corneas of mice infected with the seven different HSV-1 strains were dissected, pooled (5 corneas per pool) and corneas single cell suspensions were obtained after fine chopping followed by 1 h collagenase treatment (15 mg/ml, 37°C—Roche, Cat. No. 11088866001). Corneas infiltrated cells were then analyzed by flow cytometry after staining with the following fluorochrome-conjugated mouse-specific mAbs: anti-mouse CD4 BV510 (clone RM4-5, BioLegend), anti-mouse CD3 BV711 (clone 17A2, BioLegend), anti-mouse CD11c PE (clone HL3, BD Biosciences), anti-mouse CD11b PerCP (clone M1/70, R&D), anti-mouse F4/80 PE-cy7 (clone BM8, BioLegend), anti-mouse Ly6G APC (clone 1A8, BioLegend), anti-mouse Ly6C BV421 (clone HK1.4, BioLegend), and anti-mouse CD45 APC-Cy7 (clone 30-F11, BD Pharmingen). To define positive and negative populations, we used isotype control mAbs for each fluorophore and further optimized gating by examining known negative cell populations for background expression levels. Details of the gating strategy are described in Supplemental Figure S1A.

For surface staining, mAbs were added to a total of 2 × 10^6^ cells (in duplicate) in phosphate-buffered saline (PBS) containing 1% FBS and 0.1% Sodium azide (fluorescence-activated cell sorter [FACS] buffer) and left for 45 min on ice and in the dark. Ab capture beads (BD Biosciences) were used as individual compensation tubes for each fluorophore in the experiment.

For determination of Caspase-1 activation in leukocyte, inflammatory monocytes, and neutrophils infiltrating the corneas, FAM-YVAD-FMK (FLICA) probe (ImmunoChemistry Technologies, Cat. No. 98) was added prior the mAbs surface staining, right after corneas collagenase treatment. After 2 h incubation at 37°C with regular mixing, cells were washed three time thoroughly with Apoptosis Wash Buffer (ImmunoChemistry Technologies) followed by the surface staining for immuno-phenotyping.

For each sample of pooled corneas, all the cells were acquired on the BD FORTESA flow cytometer. Cells without FLICA treatment were used as negative control for auto-fluorescence.

### *In vitro* FLICA Staining and ASC Specks Formation Monitoring

After 24 h infection with the various HSV-1 strains (MOI of 1 or 0.1), hTCEpi cells were stained with FLICA reagent following the same already described procedure to assess Caspase-1 activation by flow-cytometry. In order to detect ASC specks formation using flow cytometry (18), we further stained hTCEpi cells intracellularly for ASC and HSV-1: cells were first treated with cytofix/cytoperm (BD Biosciences) for 30 min. Upon washing with Perm/Wash buffer, anti-ASC A647 (clone B-3, Santa Cruz Biotechnology, Santa Cruz, CA) and anti-HSV-1 (rabbit polyclonal from Abcam, Cambridge, MA, Cat. No. ab9533) were added to the cells and incubated for 45 min on ice and in the dark. After washing with Perm/Wash buffer, a goat anti-rabbit PercP-conjugated (Novus biologicals, Centennial, CO) was added for 20 additional minutes. Cells were then washed again with Perm/Wash and FACS buffer and fixed in PBS containing 2% paraformaldehyde (Sigma-Aldrich) before FACS acquisition. Details of the gating strategy are described in supplementary Figure S1B.

### Western Blotting

Infected hTCEpi cells and THP-1 cells were lysed on ice using RIPA lysis buffer supplemented with 1% protease inhibitor and the lysates were stored at −80°C for western blot analysis. After dissection, corneas were mechanically homogenized with a TissueLyser II (Qiagen) in RIPA buffer + 1% protease inhibitor. After two cycle of freezing/thawing and 10 min sonication using an ultrasonics bath (Brandson CPX 1800), cell lysates or corneas lysed homogenate were quantified using Nanodrop (ThermoFisher) and 40 μg of protein loaded to SDS-PAGE (4–15%) gel for electrophoresis (Invitrogen, Cat. No. NW04127BOX) and then transferred to a polyvinylidene fluoride (PVDF) membrane (Merck Millipore, Burlington, MA). After a 2 h blockade with 5% Bovine serum albumin (BSA), membranes were incubated with primary antibodies overnight at 4°C. To assess inflammasome proteins expression in infected human cell lines, we stained the blots with a mouse mAb anti-NLRP3 (clone D4D8T, Cell Signaling Technology, Danvers, MA), a rabbit polyclonal anti-hNLRP6 antibody (Cat. No. ABF29, EMD Millipore), a rabbit polyclonal anti-NLRP12 antibody (Cat. No. NBP1-76293, Novus biologicals), a mouse mAb anti-IFI16 (clone 1G7, Santa-Cruz Biotechnology), a rabbit polyclonal anti-hAIM2 (Cat. No. ab93015, Abcam), a mouse mAb anti-hASC (clone TMS1, MBL international), a rabbit polyclonal anti-Caspase-1 antibody (Cat. No. NBP1-45133, Novus biologicals), a rabbit mAb anti-cleaved human Caspase-1 (clone D57A2, Cell Signaling Technology), a rabbit mAb anti-IL-1β (Santa Cruz Biotechnology), a mouse anti-IL-18 antibody (clone 74801, R&D Systems Inc., Minneapolis, MN), and a mouse mAb anti- β-actin (clone AC-15, Sigma Aldrich). To determine the expression of inflammasomes in corneal lysates, we used a rabbit polyclonal anti-mouse NLRP6 (Cat. No. SAB1302240–Sigma Aldrich), a rabbit polyclonal anti-mouse p204/IFI-16 (Cat. No. ab104409, Abcam), a rabbit polyclonal anti-mouse AIM2 (p200) (Cat. No. 13095S, Cell Signaling Technology) and the same antibodies aforementioned used to stain the mouse NLRP3 and NLRP12 proteins.

Secondary antibodies included horseradish peroxidase HRP-conjugated anti-mouse IgG (Cat. No. 7076, Cell Signaling Technology) and HRP-conjugated anti-rabbit IgG (Cat. No. 7074P2, Cell Signaling Technology). Detection of protein bands was performed using enhanced chemi-luminescence (ECL; Merck Millipore, Burlington, MA) substrate and imaging with ChemiDoc imaging systems (Bio-Rad Laboratories, Hercules, CA). The immunoblots were quantified using ImageJ/Fiji and Image Lab (Bio-Rad) software and results normalized to β-actin expression.

### Statistical Analysis

Data for each assay were compared by ANOVA and Student's *t*-test using GraphPad Prism version 7 (La Jolla, CA). Differences between the groups were identified by ANOVA and multiple comparison procedures. Data are expressed as the mean ± SD. Results were considered statistically significant at *P* < 0.05.

## Results

### Ocular Infection With Virulent Strains of HSV-1 Induces Severe Corneal Herpetic Disease, Irrespective of the Level of Virus Replication in the Cornea

We first compared the severity of corneal inflammatory diseases, keratitis and blepharitis, and levels of viral replication in B6 mice following ocular infection with five HSV-1 high-passage laboratory strains (i.e., McKrae, 17, F, RE, and KOS; referred throughout the present study as “laboratory strains”) and two low-passage clinical isolates KOS79 and KOS63 (referred throughout the present study as “clinical isolates”), that presented various levels of virulence.

**Figure 1:**
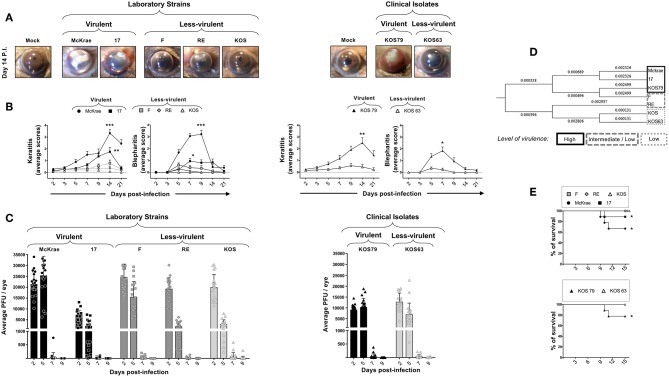
Severity of corneal inflammatory disease, viral replication, and survival in B6 mice ocularly infected with virulent and less-virulent laboratory strains and clinical isolates of HSV-1. B6 mice (*n* = 10) were infected ocularly with 2.5 × 10^5^ pfu/eye of HSV-1 laboratory strains (i.e., McKrae, 17, F, RE, or KOS) or clinical isolates (i.e., KOS63 or KOS79). HSV-1 laboratory strains and clinical isolates were divided into virulent (McKrae, 17, and KOS79, black columns), medium-virulent (F and RE, gray columns) and low-virulent (KOS and KOS63, light-gray columns). Mock-infected mice (*n* = 10) were used as controls. **(A)** Representative pictures of eye disease on day 14 post-infection from mice infected with virulent/less-virulent laboratory strains (left panels) and of virulent/less-virulent clinical isolates are shown (right panels). **(B)** Mean score keratitis and blepharitis disease scored on days 2, 3, 5, 7, 9, 14, and 21 in mice infected with virulent/less-virulent laboratory strains (left panels) and of virulent/less-virulent clinical isolates (right panels). Stromal keratitis was scored as 0- no disease; 1- cloudiness, some iris detail visible; 2- iris detail obscured; 3- cornea totally opaque; and 4- cornea perforation. Blepharitis was scored as 0- no disease; 1- puffy eyelids; 2- puffy eyelids with some crusting; 3- eye swollen shut with severe crusting; and 4- eye completely swollen shut. **(C)** Eye swabs were obtained 2, 5, 7, and 9 days post-infection and virus replication was determined. Shown are the optimal levels of replication detected on day 7 post-infection from virulent/less-virulent laboratory strains (left panels) and of virulent/less-virulent clinical isolates (right panels). **(D)** Optimal tree of a relative evolutionary distances between HSV-1 laboratory strains (i.e., McKrae, 17, F, RE, or KOS) or clinical isolates (i.e., KOS63 or KOS79) computed using the Maximum Composite Likelihood method are shown. **(E)** Percentage survival in B6 mice following ocular infection with virulent/less-virulent laboratory strains (upper panel) and clinical isolates (lower panel). *P < 0.05; **P < 0.01; ***P < 0.001 using Student's *t*-test.

The severity of corneal inflammatory disease, keratitis and blepharitis, was monitored up to 21 days post-infection. Representative pictures of corneal disease induced by each of the five laboratory strains and the two clinical isolates, taken at day 14 post-infection, can be visualized in Figure 1A. As shown in Figure 1B, severe corneal keratitis and blepharitis was detected in B6 mice following infection with the virulent HSV-1 laboratory strains McKrae and 17 (Figure 1B, left two panels) and with the virulent clinical isolate KOS79 (Figure 1B, right two panels). In contrast, mild to less-severe corneal keratitis and blepharitis was detected in B6 mice following infection with the less-virulent HSV-1 laboratory strains F, RE and KOS (Figure 1B, left two panels) and the less-virulent clinical isolate KOS63 (Figure 1B, right two panels). Therefore, strains McKrae and 17 and clinical isolate KOS79 are the most virulent, whereas strains F, RE, and KOS and clinical isolate KOS63 are the least virulent.

To determine a potential correlation of corneal disease with the amount of virus in the cornea, the level of viral replication was determined over 9 days post-infection. The HSV-1 laboratory strains McKrae, 17, F, RE, and KOS (Figure 1C, left panel) and the clinical isolate KOS63 (Figure 1C, right panel) replicated in the cornea of B6 mice as early as 2 days post-infection. In contrast, the laboratory strain 17 and the clinical isolate KOS79 exhibited a low level of replication. In both laboratory strains and clinical isolates infected animals, the virus cleared from the cornea as early as 7 days post-infection. Overall, the results indicated that the level of replication of HSV-1 laboratory strains and clinical isolates in the cornea of B6 mice does not appear to be related with the virulence level. These results indicate that the virulent HSV-1 laboratory strains McKrae and 17 and the clinical isolate KOS79 caused severe corneal inflammatory disease regardless of the level of viral replication.

The evolutionary distances, computed using the Maximum Composite Likelihood method and based on seven nucleotide sequences detected a short evolutionary distance between the virulent HSV-1 laboratory strains McKrae and 17 and the clinical isolate KOS79 (along with the F strain). Similarly, the short evolutionary distance was detected between the less-virulent laboratory strains RE and KOS and the clinical isolate KOS63. This result showed a clear difference between the higher virulent strains McKrae, 17, and KOS79 and less-virulent strains RE, KOS, and KOS63. Phylogenetic trees and genetic correlations are widely used to determine the evolutionary distances between strains of various infectious pathogens but these evolutionary distances do not always reflect the actual virulence of such pathogens (19, 20). Although the F strain falls genetically at close proximity to the most virulent strain KOS79, the F strain was less virulent following ocular infection in B6 mice. Moreover, the F strain induced a mild-severe HSK disease in B6 mice which tends to be slightly more severe than the disease induced by RE and KOS. (Figure 1B, right two panels). Further, based on the genetic distance data, KOS was evolutionarily in closer proximity to the less-virulent strains KOS63 and RE (Figure 1D).

Survival was recorded in B6 mice following infection with the less-virulent HSV-1 laboratory strains F, RE and KOS and the virulent strains McKrae, 17 (Figure 1E, top panel). Survival was also recorded in B6 mice infected with the less-virulent clinical isolate KOS63 and the virulent clinical isolate KOS79 (Figure 1E, bottom panel). Overall, the mortality was higher in the groups of mice infected with the virulent laboratory strains McKrae and 17 (Figure 1E, top panel) and the virulent clinical isolate KOS79 (Figure 1E, bottom panel).

Altogether, these results demonstrate that ocular infection with the virulent strains and virulent clinical isolate of HSV-1 induced severe corneal inflammatory herpetic disease and death irrespective of the level of viral replication.

### Inflammatory Monocytes and Neutrophils Infiltrates in Infected Cornea Following Ocular Infection With Virulent and Less-Virulent HSV-1 Strains

Inflammatory monocytes and neutrophils, contribute to corneal immunopathology during HSV-1 infection (21, 22). Inflammatory monocytes are recruited to the cornea early and peak during the acute phase, 2–5 days after infection (22) while neutrophils are recruited to the cornea later during the chronic inflammatory phase and peak 8–14 days post-infection (21).

**Figure 2:**
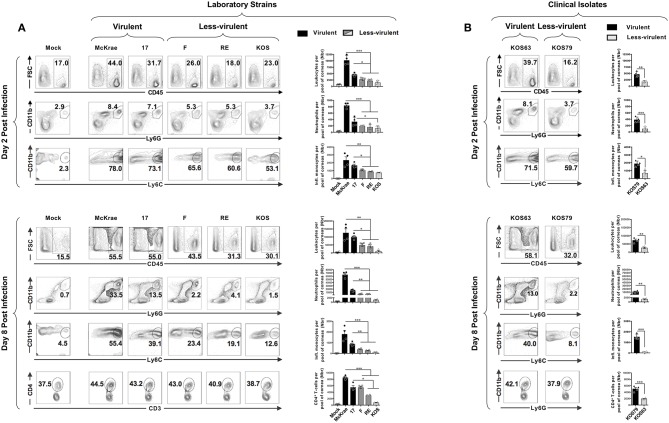
Association of inflammatory cell infiltrates to severity of corneal herpetic disease following infection with virulent and less-virulent HSV-1 strains. B6 mice (*n* = 20) were infected ocularly with 2.5 × 10^5^ pfu/eye of HSV-1 laboratory strains (i.e., McKrae, 17, F, RE or KOS) or clinical isolates (i.e., KOS63 or KOS79). Mock-infected mice (*n* = 20) were used as controls. Single corneal cell suspensions were obtained from infected and mock-infected controls on day 2 post-infection (**A,B**–upper panels) and day 8 post-infection (**A,B**–lower panels). For each time point and condition of infection, a pool of 5 corneas where chopped finely, treated with collagenase for 1 h at 37°C, and the inflammatory cells infiltrates were stained with mAbs specific for CD45, CD11b, CD11c, Ly6G, Ly6C, F4/80, CD3, and CD4^+^, and analyzed by FACS. (**A,B**–upper panels) FACS plots of leukocytes (CD45^high^), inflammatory monocytes (CD45^high^Ly6C^+^Ly6G^−^F4/80^+^CD11b^+^CD11c^−^) and neutrophils (CD45^high^CD11b^+^F4/80^−^Ly6G^hi^) infiltrates in cornea of mice 2-day post-infection with virulent (McKrae, 17) and less-virulent (F, RE and KOS) laboratory strains **(A)** and with virulent (KOS79) and less-virulent (KOS63) clinical isolates **(B)**. (**A,B**–lower panels) FACS plots of leukocytes (CD45^high^), inflammatory monocytes (CD45^high^Ly6C^+^Ly6G^−^F4/80^+^CD11b^+^CD11c^−^), neutrophils (CD45^high^CD11b^+^F4/80^−^Ly6G^hi^), and CD3^+^CD4^+^ T cell infiltrates in cornea of mice 8-day post-infection with virulent (McKrae, 17) and less-virulent (F, RE, and KOS) laboratory strains **(A)** and with virulent (KOS79) and less-virulent (KOS63) clinical isolates **(B)**. FACS dot plots show representative results (with percentages) and the column graphs represent the average absolute cell numbers (± SEM). Those results are representative of 3 independent experiments. The gating strategy is described Supplemental Figure S1A. *P < 0.05; **P < 0.01; ***P < 0.001, using ANOVA-multiple-comparison-test.

We next determined the frequency of pan CD45^high^ leukocytes, CD45^high^Ly6C^+^Ly6G^−^F4/80^+^CD11b^+^CD11c^−^ inflammatory monocytes, CD45^high^CD11b^+^F4/80^−^Ly6G^hi^Ly6C^med^ neutrophils, and CD3^+^CD4^+^ T cells infiltrating the inflamed cornea at day 2 and day 8 post-infection with virulent vs. less-virulent laboratory strains and clinical isolates of HSV-1. The size of immune cell infiltrates was correlated with the severity of corneal inflammatory disease. Two days post-infection of B6 mice with virulent strains (McKrae, 17), the cornea appeared infiltrated with acute inflammatory CD45^high^ leukocytes with a majority of Ly6C^+^Ly6G^−^F4/80^+^CD11b^+^CD11c^−^ inflammatory monocytes and CD11b^+^F4/80^−^Ly6G^hi^Ly6C^med^ neutrophils (Figure 2A, upper left and right panels). Similarly, at day 2 post-infection of B6 mice with the virulent clinical isolate KOS79, the percentages (Figure 2B, upper left panels) and the absolute numbers (Figure 2B, upper right panels) of inflammatory monocytes and neutrophils were significantly increased in the cornea. At day 8 post-infection, corneal infiltration with CD11b^+^F4/80^−^Ly6G^hi^ neutrophils was even higher (Figures 2A, B, lower panels). Compared to day 2 post-infection, on day 8 post-infection, the difference in the frequency of CD11b^+^F4/80^−^Ly6G^hi^ neutrophils that infiltrate the cornea of B6 mice infected with virulent strains (McKrae, 17, KOS79) vs. those that infiltrate the cornea of B6 mice infected with less-virulent strains (F, RE, KOS, KOS63) was even significantly larger (Figures 2A, B, lower panels). Moreover, at day 8 post-infection, we observed a significant difference in the number of CD3^+^CD4^+^ T cells that infiltrated the cornea of B6 mice infected with the virulent vs. less-virulent strains. Overall, the larger size of CD45^high^ Ly6C^+^Ly6G^−^F4/80^+^CD11b^+^CD11c^−^ inflammatory monocytes and CD45^high^CD11b^+^F4/80^−^Ly6G^+^ neutrophils infiltrates induced by virulent strains (McKrae, 17, KOS79) correlated with the severity of corneal inflammatory herpetic disease.

When comparing the frequencies and numbers of neutrophils on day 2 (Figures 2A, B, upper panels – second rows), vs. day 8 post-infection (Figures 2A,B, lower panels – second rows), we observe a significant increase of neutrophils overtime in the corneas of mice infected with the virulent laboratory strains and the virulent clinical isolate (average increase of ~80-fold with McKrae; ~35-fold with strain ~17; and ~30-fold with KOS79–Supplemental Figure S2A). In contrast, the less virulent strains and the clinical isolate only induced a moderate neutrophil infiltration overtime (average increase of ~24-fold with F strain; ~28-fold with RE; ~11-fold with KOS; ~13-fold with KOS63–Supplemental Figure S2A). On the other hand, comparison of the frequency of inflammatory monocytes on day 2 (Figures 2A,B, upper panels–second rows), vs. day 8 post-infection (Figures 2A,B, lower panels – second rows), showed a decrease on day-8 compared to day 2 for all the strains (Supplemental Figure S2B). However, the decrease of inflammatory monocytes from day 2 to day 8 was less significant with virulent strains: ~1.3-fold for McKrae; 1.9-fold for strain 17; 1.2-fold for KOS79 whereas the average decrease was 3 to 6-fold for less virulent strains (Supplemental Figure S2B).

Altogether, these results indicate that the HSV-1 virulence promotes a rapid elevated recruitment/infiltration into the inflamed cornea of CD45^high^Ly6C^+^Ly6G^−^F4/80^+^CD11b^+^CD11c^−^ inflammatory monocytes early during the acute phase and of CD45^high^CD11b^+^ F4/80^−^Ly6G^hi^ neutrophils late during the chronic inflammatory phase, and this was associated with severe corneal inflammatory disease. The results also suggest that inflammatory monocytes were either (i) retained longer in the corneas infected with the virulent strains compared to less-virulent strains; or (ii) inflammatory monocytes infiltrate more on day 8 in the corneas infected with the virulent strains compared to less-virulent strains.

### Virulent Strains of HSV-1 Selectively Activate Caspase-1 in Both Inflammatory Monocytes and Neutrophils Infiltrating the Inflamed Corneas With Severe Herpetic Disease

Caspase-1 activation is an important early innate inflammatory response to infection by viral pathogens. We next used the FLICA assay to assess the level of Caspase-1 activation in inflammatory monocytes and neutrophils infiltrating the inflamed cornea of B6 mice infected with virulent (McKrae, 17 or KOS79) and less-virulent (KOS, RE, F or KOS63) strains and clinical isolates of HSV-1.

**Figure 3:**
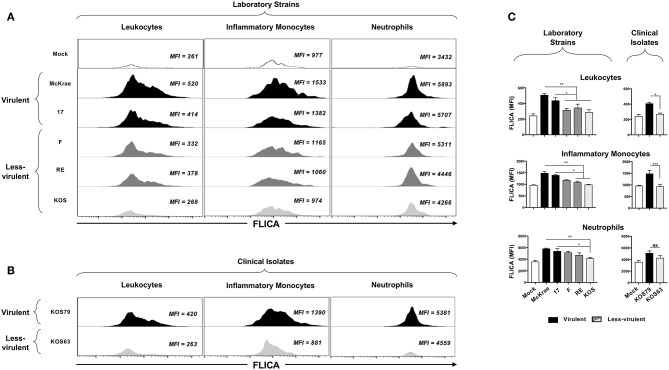
FLICA upregulation (i.e., Caspase-1 activation) in immune cells infiltrating the cornea infected with virulence and less-virulence strains of HSV-1. B6 mice (*n* = 5) were infected ocularly with 2.5 × 10^5^ pfu/eye of HSV-1 laboratory strains (i.e., McKrae, 17, F, RE or KOS) or clinical isolates (i.e., KOS63 or KOS79). Mock-infected mice (*n* = 5) were used as controls. Single corneal cell suspensions were obtained from infected and mock-infected controls on day 2 post-infection (2 pools of 5 corneas were acquire in duplicate). **(A)** FLICA expression levels in cornea-derived leukocytes (left panels), inflammatory monocytes (middle panels) and neutrophils (right panels) 2-day post-infection with virulent (McKrae, 17) and less-virulent (F, RE, and KOS) laboratory strains are shown. **(B)** FLICA expression levels in cornea-derived leukocytes (left panels), inflammatory monocytes (middle panels) and neutrophils (right panels) 2-day post-infection with virulent (KOS79) and less-virulent (KOS63) clinical isolates are shown. **(C)** Column graphs represent the average percentages and absolute cell numbers from three independent experiments (± SEM). *P < 0.05; **P < 0.01; ***P < 0.001, using ANOVA-multiple-comparison-test.

Representative histogramsofactivatedCaspase-1intotalleukocytes(CD45^high^); inflammatorymonocytes (CD45^high^Ly6C^+^Ly6G^−^F4/80^+^CD11b^+^CD11c^−^); and neutrophils(CD45^high^CD11b^+^ F4/80^−^Ly6G^hi^) in the cornea infected with virulent (McKrae, 17 or KOS79) and less-virulent (KOS, RE, F or KOS63) strains and clinical isolates of HSV-1 is shown in Figures 3A–C. Overall, there was an increase of FLICA (i.e., Caspase-1 activation) in CD45^+^ Leukocytes infiltrating the corneas infected with virulent HSV-1 laboratory strains (McKrae and 17) (Figure 3A, upper panels) and with the virulent clinical isolate KOS79 (Figure 3B, upper panel). In contrast, less significant increase FLICA was detected in CD45^+^ leukocytes infiltrating the corneas infected with less-virulent HSV-1 laboratory strains (F, RE and KOS) (Figure 3A, lower panels) and less-virulent clinical isolate KOS63 (Figure 3B, lower panel). Similar trends of high activation of Caspase-1 (increase of FLICA) were detected in inflammatory monocytes (CD45^high^Ly6C^+^Ly6G^−^F4/80^+^CD11b^+^CD11c^−^) and neutrophils (CD45^high^CD11b^+^ F4/80^−^Ly6G^hi^) infiltrating the cornea of B6 mice infected with virulent strains of HSV-1 (Figures 3A,B). The average of FLICA activation depicted by median fluorescent intensity (MFI) from two pools of 5 corneas of 5 infected mice showed a significant increase in activated Caspase-1 in leukocytes (CD45^high^); inflammatory monocytes (CD45^high^Ly6C^+^Ly6G^−^F4/80^+^CD11b^+^CD11c^−^); and neutrophils (CD45^high^CD11b^+^ F4/80^−^Ly6G^hi^) (Figure 3C).

Altogether, these results indicate that infection with virulent strains of HSV-1 promoted Caspase-1 activation in the inflammatory monocytes and neutrophils infiltrating the inflamed corneas.

### Virulent HSV-1 Strains Induce the NLRP3, NLRP12, and IFI16/p204 Inflammasomes in Mouse Cornea

**Figure 4:**
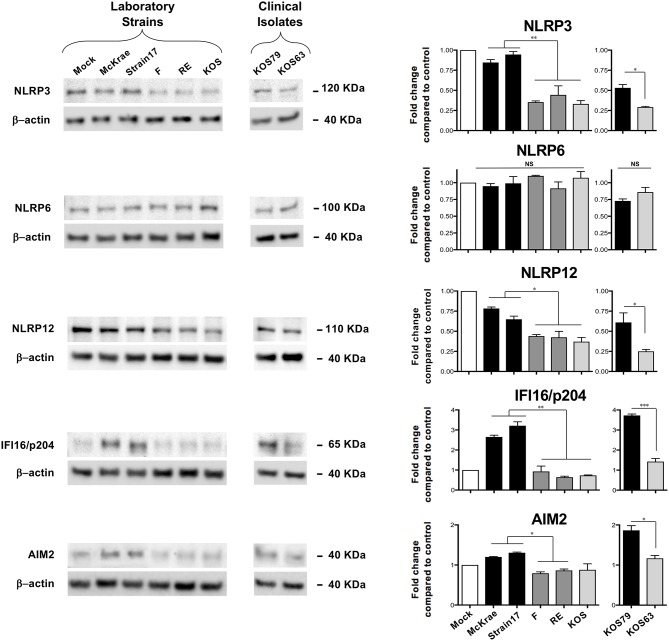
Expression levels of NLRP3, NLRP6, NLRP12, IFI16/p204, and AIM2 inflammasomes in mouse corneas following infection with virulent and less-virulent strains of HSV-1. B6 mice (*n* = 6) were infected ocularly with 2.5 × 10^5^ pfu/eye of HSV-1 laboratory strains McKrae, 17, F, RE or KOS or with clinical isolates KOS79 and KOS63. Mock-infected mice (*n* = 6) were used as controls. Forty-eight hours post-infection animals were euthanized the corneas were excised and homogenized in lysis buffer, and western blot was performed for expression of NLRP3, NLRP6, NLRP12, IFI16/p204, and AIM2. Corresponding β-actin was used as a control. Three western blot were performed using three different corneal lysates harvested from three sets of corneas pooled from 2 infected mice (i.e., 4 corneas per lysate). Results are representative of those three independent experiments. Graphs (mean plus SD) show fold change in expression of NLRP3, NLRP6, NLRP12, IFI16/p204, and AIM2 at 48 h post-infection with virulent/less-virulent strains of HSV-1 compared to the control and normalized to β-actin. Representative densitometric analysis is shown compared to control. *P < 0.05; **P < 0.01; ***P < 0.001 using Student's *t* test.

Since induction of inflammasomes triggers Caspase-1 activation, we next determined whether corneal infection with virulent HSV-1 strains would enhance the expression levels of one or several of the five major inflammasomes: NLRP3, NLRP6, NLRP12, IFI16/p204, and AIM2. Although mice do not express an IFI16 gene, they express a close ortholog of IFI16, designated IFI16/IFI204 or IFI16/p204. Several studies reported that the mouse IFI16/p204 ortholog has a similar structure and do functions as the human IFI16 (23–27). In this study, we detected a protein at 65KD molecular weight (Figure 4), which is similar to the reported 65/70KD molecular weight of the mouse IFI16/p204 ortholog. B6 mice were infected with virulent vs. less-virulent laboratory strains and clinical isolates of HSV-1 and the levels of these inflammasomes were examined by western blot in mouse corneal lysates 48 h post-infection. As shown in Figure 4, levels the levels of expression of NLRP3, NLRP12, AIM2, and IFI16/p204 inflammasomes were significantly higher in the corneas of mice infected with the virulent (McKrae, 17 or KOS79) compared to less-virulent (KOS, RE, F or KOS63) strains of HSV-1 (*P* < 0.05). In contrast, expression levels of the NLRP6 inflammasome were similar in the cornea of mice B6 infected with virulent and less-virulent strains.

These results confirm that infection with virulent HSV-1 strains and clinical isolate induced the expression of NLRP3, NLRP12, AIM2, and IFI16/p204 inflammasomes, but not of the NLRP6 inflammasome.

### Selective and Early Activation of NLRP3, NLRP12, and IFI16 Inflammasomes in Human Corneal Epithelial Cells and THP-1 Cells Following Infection With Virulent HSV-1 Strains

Since epithelial cells are the first corneal cells targeted by HSV-1, we next determined whether and how the NLRP3, NLRP6 and NLRP12, IFI16, and AIM2 inflammasomes pathways are activated in human corneal epithelial cells following *in vitro* infection with virulent vs. less-virulent laboratory strains and clinical isolates of HSV-1.

**Figure 5:**
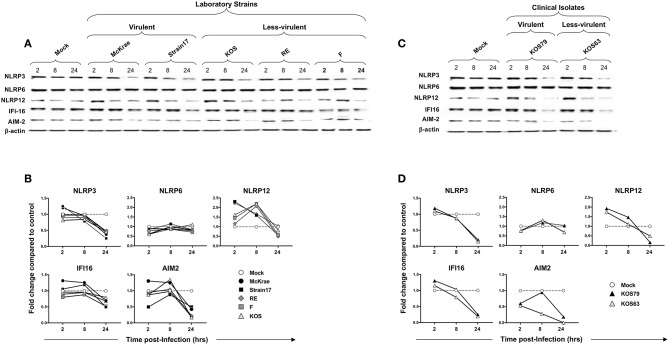
Expression level of NLRP3, NLRP6, NLRP12, IFI16 and AIM2 inflammasomes in human primary corneal epithelial cells infected with virulent and less-virulent strains of HSV-1. Human primary corneal epithelial cells (hTCEpi cell line) were infected *in vitro* with HSV-1 laboratory strains McKrae, 17, F, RE or KOS **(A,B)** or with clinical isolates KOS79 and KOS63 **(C,D)** at MOI of 1. Two, 8 and 24 h post-infection, hTCEpi cells were harvested and immunoblots of whole cell lysates were performed for expression of NLRP3, NLRP6, NLRP12, IFI16, and AIM2 inflammasomes. Corresponding β-actin was used as a control. **(B,D)** Graphs show the kinetics of fold changes in NLRP3, NLRP6, NLRP12, IFI16, and AIM2 inflammasomes expression at 2, 8, and 24 h post-infection with virulent/less-virulent strains of HSV-1 compared to the control and normalized to β-actin. Results are representative of three experiments.

Human corneal epithelial hTCEpi cells were infected with virulent (McKrae, 17 or KOS79) and less-virulent (KOS, RE, F, or KOS63) strains of HSV-1 at MOI of 1. Cells were harvested at 2, 8, and 24 h post-infection and the level of expression of NLRP3, NLRP6, NLRP12, IFI16, and AIM2 was determined by western blot. Infection of human corneal epithelial cells with virulent HSV-1 laboratory strains (McKrae or 17) (Figures 5A,B) or with the clinical isolate KOS79 (Figures 5C,D) induced a significant early expression of NLRP3 and IFI16 inflammasomes, compared to a steady level of control β-actin (*P* < 0.05). In contrast, infection with less-virulent HSV-1 strains (KOS, RE or F) or with the clinical isolate KOS63 induced a lower expression level of NLRP3 and IFI16 inflammasomes. The level of expression of NLRP6 inflammasome remained similar following infection with virulent and less-virulent strains of HSV-1.

At 2 and 8 h (but not at 24 h) post-infection of hTCEpi cells, all strains, regardless of virulence, induced expression of NLRP12 at a level higher than mock. However, compared to less-virulent strains, the virulent strains induced higher activation of NLRP12 at 2 h post-infection of hTCEpi cells. Similar to hTCEpi cells, high levels of inflammasomes expression were induced in THP-1 cells by virulent HSV-1 strains (17, McKrae, and KOS79) (Supplemental Figure S3). Moreover, the expression levels of NLRP3, NLRP12 and IFI-16 was sustained longer (i.e., until 24 h) in THP-1 cells after infection with the virulent strains. Furthermore, similar to hTCEpi cells, lower levels of inflammasomes expression were detected in THP-1 cells infected less-virulent HSV-1 strains (F, RE, KOS, and KOS63). Unlike hTCEpi cells, THP-1 cells infected by the virulent strains also expressed higher levels of NLRP6.

These results suggest that, unlike the less-virulent, infection of human corneal epithelial cells and THP-1 cells with virulent strains, induced simultaneous and multiple activations of NLRP3, NLRP12, and IFI16 inflammasomes.

### Cleaved Caspase 1, Cleaved IL-1β, and IL-18 Are Produced by Human Corneal Epithelial and THP-1 Cells Following Infection With Virulent Strains of HSV-1

Having demonstrated that infection with virulent HSV-1 strains increased NLRP3, NLRP12, and IFI16 inflammasome in infected human corneal epithelial cells, we next determined the level of expression of several components downstream of these inflammasomes. Immunoblots of whole lysates of hTCEpi human corneal epithelial cells infected with virulent and less-virulent strains of HSV-1 at 1 MOI, were probed for inflammasome speck component ASC (adaptor protein), pro- and cleaved Caspase-1 (also known as ICE, IL-1β converting enzyme), pro- and cleaved IL-1β and IL-18.

**Figure 6:**
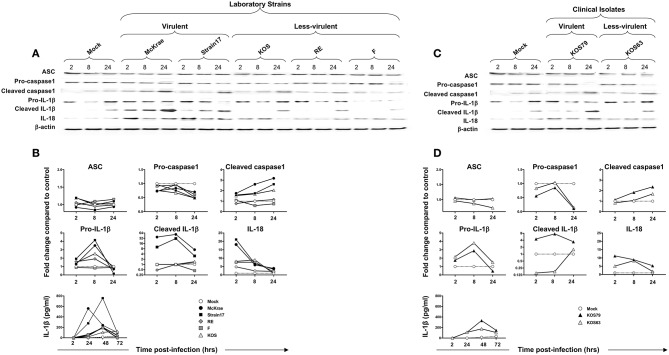
Expression levels of the adapter protein ASC, Caspase-1, IL-1β, and IL-18 in human primary corneal epithelial cells infected with virulent and less-virulent strains of HSV-1. Human primary corneal epithelial cells (hTCEpi cell line) were infected *in vitro* with HSV-1 laboratory strains McKrae, 17, F, RE or KOS **(A,B)** or with clinical isolates KOS79 and KOS63 **(C,D)** at MOI of 1. Four, 8 and 24 h post-infection, hTCEpi cells were harvested and immunoblots of whole cell lysates were performed for expression of ASC (adaptor protein), Caspase-1 (also known as ICE, IL-1β converting enzyme), IL-1β and IL-18. Corresponding β-actin was used as a control. IL-1β was also measured in culture supernatants by ELISA. **(B,D)** Graphs show the kinetics of fold changes in expression of ASC (adaptor protein), Caspase-1, IL-1β and IL-18 at 2, 8, and 24 h post-infection with virulent/less-virulent strains of HSV-1 compared to the control and normalized to β-actin. Results are representative of three experiments.

**Figure 7:**
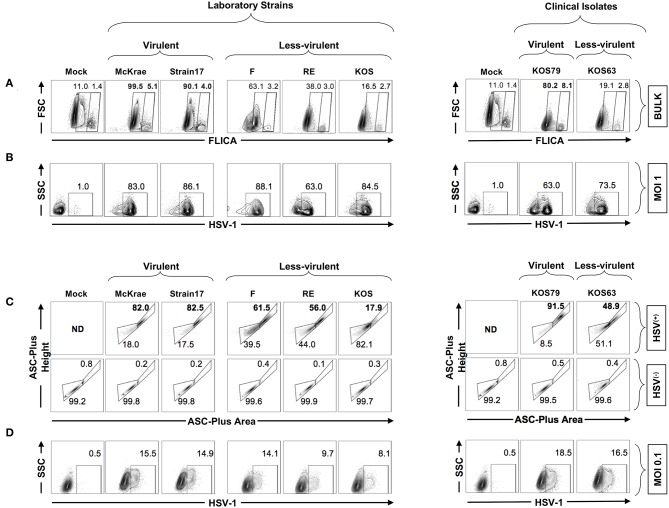
Formation of single ASC specks in HSV^(+)^ and HSV^(−)^ infected human corneal epithelial cells infected with virulent and less-virulent strains of HSV-1. Human primary corneal epithelial cells (hTCEpi cell line) were infected *in vitro* with HSV-1 laboratory strains McKrae, 17, F, RE or KOS or with clinical isolates KOS79 and KOS63 at MOI of 1 **(A,B)** or MOI of 0.1 **(C,D)**. Twenty-four hours post-infection, cells were harvested and stained for FAM-FLICA and analyzed by FACS to determine the levels of expression of single and dense ASC specks, a prerequisite for to Caspase-1 activation. **(A)** Contour plots showing percentage of FAM-FLICA^+^ (activated Caspase-1^+^ cells) expressed in bulk cells. **(B)** Contour plots showing percentage of infected cells (HSV-1^+^ cells) at (MOI = 1). **(C)** Contour plots showing the ASC aggregation (comparing ASC-height vs. ASC-area) in HSV-1^(+)^ cells (upper panels) or in uninfected bystander cells (HSV-1^(−)^ (lower panels). **(D)** From the same culture: contour plots showing percentage of infected cells (HSV-1^+^ cells) at (MOI = 0.1). The gating strategy is described in Supplemental Figure S1B. Results are representative of three experiments.

Although ASC expression detected by western blot was not increased in hTCEpi cells following infection with virulent strains, the ASC was re-localized into a speck (**Figure 7C**), confirming a previous report (18). The cleaved/activated Caspase-1, cleaved/activated IL-1β, and IL-18 were significantly induced in human corneal epithelial cells following infection with virulent laboratory strains of HSV-1, McKrae and 17, as early as 2 h post-infection, compared to control β-actin (*P* < 0.05) (Figures 6A,B). In contrast, the less-virulent strains KOS, RE and F induced significantly lower levels of cleaved/activated Caspase-1, cleaved/activated IL-1β and IL-18 (*P* < 0.05). Similarly, infection of human corneal epithelial cells with virulent HSV-1 clinical isolate KOS79 (Figures 6C,D) induced a significant early activation of Caspase-1, IL-1β and IL-18. In contrast, infection with less-virulent HSV-1 clinical isolate KOS63 induce less activation of Caspase-1, IL-1β and IL-18. Although all viruses increased the levels of IL-18 in hTCEpi cells compared to mock-infected hTCEpi cells, the virulent HSV-1 strains (17, McKrae, and KOS79) induced significantly higher levels of IL-18 early at 2 h post-infection compared to less-virulent HSV-1 strains (F, RE, KOS, and KOS63) (Figures 6A–D). Finally, high-levels of IL-1β were detected by ELISA in the supernatant of infected cells as early as 4 h post-infection with virulent laboratory strains of HSV-1, McKrae, and 17 and the virulent clinical isolate KOS79 (Figure 6B, bottom panel). In contrast, non-significant levels of IL-1β were detected by ELISA in the supernatant of cells infected with less-virulent KOS, RE and F laboratory strains and the less-virulent clinical isolate KOS63.

Similar to hTCEpi cells, high levels of cleaved Caspase 1, cleaved IL-1β, and IL-18 were induced by the virulent HSV-1 strains (17, McKrae, and KOS79) in THP-1 cells. Caspase-1, IL-1β activations and IL-18 production seemed to be even faster and sustained longer in THP-1 cells (Supplemental Figure S3). Moreover, similar to hTCEpi cells, low levels of cleaved Caspase 1, cleaved IL-1β and IL-18 were induced by the less-virulent HSV-1 strains (F, RE, KOS, and KOS63) in THP-1 cells (Supplemental Figure S3).

Altogether, these results indicate that virulent strains of HSV-1 induce more inflammasome formation causing increased activation of cleaved Caspase-1 in turn promoting cleavage of pro-IL-1β into cleaved IL-1β and the production of the biologically active pro-inflammatory cytokine IL-1β in both hTCEpi cells and THP-1 cells.

### The HSV-1 Virulence Promotes the Formation of ASC Specks and Caspase-1 Activation in HSV^(+)^ Infected Human Corneal Epithelial Cells, but Not in the Neighboring Bystander Non-infected HSV^(−)^ Cells

We next used the FLICA Caspase-1 assay to validate the previous western blot results and confirm whether Caspase-1 is more activated in human corneal epithelial following infection with virulent strains and clinical isolate of HSV-1. Human corneal epithelial hTCEpi cells were infected for 24 h with McKrae, 17, F, RE or KOS laboratory strains and with KOS63 or KOS79 clinical isolates at MOI of 1. The frequencies of FLICA^(+)^ hTCEpi cells, with Caspase-1 activation, was determined by FACS in cells infected with virulent vs. less-virulent strains (Figure 7A). In parallel, cells were stained with a polyclonal antibody specific to HSV-1 antigens and the frequency of HSV-1(+) infected cells was determined (Figure 7B).

As shown in Figure 7A, infection of human corneal epithelial cells with virulent HSV-1 strains (McKrae or 17) or with the clinical isolate KOS79 induced a significant increase of the FLICA straining, confirming the western blot data that Caspase-1 is highly activated in human corneal epithelial by virulent strains. In contrast, infection with the less-virulent HSV-1 strains (KOS, RE or F) or with the clinical isolate KOS63 induced less FLICA confirming a lower Caspase-1 activation by the less-virulent strains. There was no positive correlation between the frequencies of FLICA^(+)^hTCEpi cells and HSV^(+)^hTCEpi cells (Figures 7A,B). This supports the *in vivo* results also showing the lack of positive correlation between Caspase-1 activation (i.e., frequencies of FLICA^(+)^ corneal cells) and the level of virus replication in the cornea.

We next asked how inflammasomes are activated within a mixed population of infected (HSV^(+)^) and non-infected (HSV^(−)^) cells. Central to most inflammasome activation is the adapter molecule ASC (apoptosis-associated speck-like protein containing a Caspase-recruitment domain) that links the inflammasome initiator protein to the recruited Caspases. ASC is normally diffused within the cytosol but within minutes of inflammasome activation, it reorganizes to a few dense specks. The dramatic redistribution of diffuse ASC into one or few specks can be monitored by flow cytometry using parameters of fluorescence peak height and area (or height and width) when immuno-stained (18, 28). We therefore used a time of flight inflammasome evaluation assay, to detect potential changes in ASC distribution within the mixed population of infected (HSV^(+)^) and neighboring non-infected (HSV^(−)^) human corneal epithelial cells (Figure 7C,D–gating strategy is shown Supplemental Figure S1B). Following infection with virulent HSV-1 strains (McKrae or 17) or with the clinical isolate KOS79, the human corneal epithelial cells stained strongly for ASC, and did form dense ASC specks. In contrast, after infection with the less-virulent HSV-1 strains (KOS, RE, or F) or with the less-virulent clinical isolate KOS63, the human corneal epithelial cells stained moderately for ASC, but did not form dense ASC specks (Figure 7C). Moreover, the vast majority of HSV^(+)^ infected human corneal epithelial cells within the same mixed cell culture displayed a striking transition to the high H:A profile (Figure 7C, upper panels). In contrast, the neighboring bystander non-infected HSV^(−)^ cells did not display a transition to the high H:A profile suggesting a lack of inflammasome activation (Figure 7C, lower panels).

Altogether, these results indicate that HSV-1 virulence promotes the formation of dense ASC specks that lead to Caspase-1 activation in HSV^(+)^ infected human corneal epithelial cells, but not in the neighboring bystander non-infected HSV^(−)^ cells, suggesting that actual cells infection is a pre-requisite of inflammasome activation.

## Discussion

The host fights viral pathogens using innate and adaptive immune mechanisms (29, 30). Early during viral infections, canonical, and non-canonical inflammasomes detect virulent and less-virulent virus pathogens orchestrating the innate and adaptive immunity to clear the infection and cure the disease [reviewed in Sutterwala et al. (31) and Elliott and Sutterwala (32)]. Activation of inflammasomes by viral factors triggers maturation of proinflammatory cytokines and chemokines that function to recruit immune cells, such as neutrophils, dendritic cells, inflammatory monocytes and CD4^+^ T cells, to the site of viral infection (8, 9). This optimal activation of inflammasomes is highly beneficial to the well-being of the host as it helps the innate and adaptive immune system in clearing the infection and/or curing the ensuing disease (5–7). However, beside its protective role, an aberrant activation of inflammasomes can lead to a pathogenic inflammatory response (33). This outcome depends on both the type and the level of activation of the host's inflammasomes and the involved viral virulence factors (34, 35). Thus, the activation of inflammasomes to fighting viral pathogens can sometimes lead to tissue damage and leave casualties in the battlefield. Dysregulation or hyper-activation of the inflammasomes by virulent strains of a virus can lead to an exaggeration of inflammatory responses that lead to an exacerbation in the symptoms of viral diseases (5–7, 36). Although intensive research efforts are focusing on achieving an integrated view about the protective/detrimental role of inflammasomes in the maintenance of infected tissue integrity, only a few reports have paid attention to the role of viral virulence and virulent factors in the inflammasome activation. Moreover, we characterized the type of inflammatory cells infiltrate in the inflamed cornea that was triggered by multiple inflammasomes following ocular infection with various strains and clinical isolates of HSV-1. A positive correlation between quantitative and qualitative expression of inflammasomes, on one hand, and the severity of inflammatory corneal herpetic disease, on the other hand, is reported.

Both virulent and less-virulent strains of HSV-1 engaged the same types of inflammasomes (instead of different types) but, the levels of inflammasome activation was different, leading to either a protective or harmful inflammatory response. Thus, infection of the cornea with virulent strains of HSV-1 appeared to deregulate or induce high levels of inflammasome activation leading to a “harmful” inflammatory and severe stromal corneal disease. In contrast, the less-virulent strains of HSV-1 appeared to lead to an optimal inflammasome activation that is beneficial to the well-being of the cornea. The induced corneal lesions occurred irrespective of the levels of virus replication in the cornea. This confirms previous reports that lesions in the corneal stroma are largely attributable to inflammatory events (37). The activation of NLRP3, NLRP12, and IFI16 inflammasomes preferentially by virulent HSV-1 strains would be mediated by yet-to-be determined virulent factors. We report here that early during infection, HSV-1 seems simultaneously sensed by multiple inflammasomes and that the virulence of the virus determines the increased expression of different inflammasomes triggers and the level of inflammasome (Caspase-1) activation along with the subsequent clinical and sub-clinical inflammation. Previous reports showed that infection with VZV, another alphaherpes virus, activated NLRP3 inflammasome independent of AIM2 protein (38). Therefore, it remains to be determined whether or not the observed activation of multiple NLRP3, NLRP12, and IFI16 Inflammasomes is a hallmark of HSV-1 infection, while the role of other inflammasomes are yet to be determined.

Among the 20 types of the inflammasome complexes that have been reported thus far, the NLRP3 inflammasome is the most thoroughly studied [reviewed in Sutterwala et al. (31)]. NLRP3 inflammasome has also been the most reported in herpes infections, with consequent Caspase-1 and IL-1β activation (35, 39). A previous report also showed that HSV-1 activates NLRP3 inflammasome and therefore plays a protective role against viral immunopathological corneal lesions (37). The present study extends this report by suggesting that activation of the NLRP3 inflammasome is strain dependent. Besides, NLRP3, the IFI16, also known as interferon-inducible myeloid differentiation transcriptional activator, appeared to also be activated. Chandran et al., recently showed that BRCA1 regulates IFI16-mediated nuclear innate sensing of herpes viral DNA and subsequent induction of the innate inflammasome and interferon-β responses (35, 40). Early recognition of herpes by the IFI16 inflammasome induces acetylation of nuclear IFI16 and is essential for its cytoplasmic translocation and inflammasome responses (41). An involvement of the autophagy mechanism is not excluded as intersections between the autophagy and inflammasome pathways have been observed (42). Ocular infection of NLRP3 deficient mice led to more-severe and earlier stromal keratitis lesions and had higher angiogenesis scores than did infected wild-type animals. In addition, NLRP3^−/−^ mice generated an increased early immune response with heightened inflammatory chemokines and cytokines, including IL-1β and IL-18, and an elevated recruitment of neutrophils. Increased numbers of CD4^+^ T cells were seen at later stages of the disease in these NLRP3^−/−^ animals. The beneficial role of NLRP reported here might be related to the low level of NLRP induced by the less-virulent RE strain used in the study (37) as compared to a much higher level that would be induced by other more virulent strains such as the McKrae and 17 strains. Indeed, a side-by-side comparison of the less-virulent RE strain with the virulent McKrae and 17 strains showed the former induced a less harmful sub-clinical corneal inflammation whereas the latter induced a more “harmful” excessive inflammation that led to corneal damage. Johnson et al., showed that early on during *in vitro* infection of human foreskin fibroblasts (2 to 4 h), HSV-1 induced the activation of the IFI16 and NLRP3 inflammasomes and maturation of IL-1β (35).

In the present study, induction at a high-level of the nucleotide-binding domain leucine-rich repeat containing receptor family member (NLRP12) by virulent strains of HSV-1 (McKrae, 17, and KOS79) and increased neutrophils and dendritic cells recruitment into inflamed cornea were associated with severe corneal inflammatory disease. The NLRP12 inflammasome was recently reported by both Sutterwala and Cassel groups to mediate adverse neutrophilic recruitment during lethal influenza virus infection. NLRP12 acts as a positive activator of inflammation in other systems (36, 43–45) and a negative regulator of inflammation in many other systems (46). Whether NLRP12 deficient mice are protected from corneal HSV-1 infection and disease, compared to wild type B6 mice, is currently being investigated. Dysregulation or hyper activation of the inflammasomes following a viral infection can lead to an exaggeration of inflammatory responses that lead to exacerbation in the symptoms of viral diseases (5–7, 36). NLRP12 up-regulates MHC-I expression (47) and downregulates NFκB activation and TLR signaling in certain contexts (47). NLRP12 is highly expressed in neutrophils and DCs, and mice deficient in NLRP12 had reduced inflammatory responses, as the NLRP12^−/−^ DCs were hindered in their ability to migrate to draining LNs (48). A reduced inflammatory response in these models was not a result of defective antigenic presentation or inflammasome activation (48). In addition, a role for NLRP12 as a negative regulator of inflammation (49), similar to NLRP6, has been reported. NLRP12^−/−^ mice were unable to down-regulate NFκB and ERK activation in macrophages (49).

The AIM2 inflammasome, a key cytosolic signaling complex activated by double-stranded DNA viruses, was also engaged early after viral infections (50, 51). However, implication of AIM2 in sensing HSV-1 appears controversial. On one hand, it was reported that an AIM2-independent pathway, HSV-1 still induced strong Caspase-1 activation in the human and mouse macrophages and primary human fibroblasts (35, 52). On the other hand, one recent study indicated that AIM2 is essential for host defense against cytosolic DNA viruses, such as HSV-1 (4). As an immune evasion strategy, HSV-1 appears to use its VP22 tegument protein to inhibit AIM2-dependent inflammasome activation to enable efficient viral replication (53). We discovered that the McKrae and KOS strains, but not the F strain, induced a significant level of AIM2 in human primary corneal epithelial cells, at 4 and 8 h post-infection, however AIM2 expression faded by 24 h. The apparent differences between our study and previous reports may be attributed to different cell lines used. Interestingly the most virulent HSV-1 strain 17 inhibited AIM2 inflammasome in corneal epithelial cells as early as 4 h PI and this inhibition remains up to 24 h. This result suggests that at least some virulent strains do interfere with the AIM2 pathway to dampen the host immune response as an immune evasion mechanism and this leads to severe corneal inflammatory herpetic disease. Our results are in agreement with previous reports demonstrating that HSV-1 inhibits AIM2-dependent inflammasome activation to enable efficient viral replication (51, 53). Although AIM2 is an important DNA sensor and mediator of the inflammasome response to some DNA viruses, this PRR is probably not involved in the recognition of all strains and clinical isolates of HSV-1.

Finally, besides the AIM2, NLRP3 and NLRP12 inflammasomes, we also found high levels of activated IFI16as early as 2 h post-infection by the virulent HSV-1 strains and clinical isolates (i.e., McKrae, 17, and KOS79), and interestingly subsequent inhibition, by a yet-to-be determined immune evasion mechanism, leading to severe corneal inflammatory disease. This result extends a previous report showing that HSV-1 infection induces activation and subsequent inhibition of the IFI16 inflammasome (35, 54). Chandran et al., recently showed that *BRCA1* regulates IFI16-mediated nuclear innate sensing of herpes viral DNA and subsequent induction of the innate inflammasome and interferon-β responses (35, 40). Herpes virus genome recognition induces acetylation of nuclear IFI16 and is essential for its cytoplasmic translocation and inflammasome responses (41). IFI16 is a member of the PYHIN family and predominantly localized to the nucleus, but a pool of the intracellular IFI16 also localizes to the cytoplasm (27, 49, 55–57). IFI16-mediated sensing of HSV-1 DNA takes place independently of the nuclear entry of viral DNA in monocyte-derived macrophages, in which IFI16 co-localizes with HSV-1 DNA in the cytosol (27). In addition to signaling an innate inflammatory response upon HSV-1 infection, IFI16 also acts as a restriction factor for immediate-early gene (IE) expression of HSV-1 by promoting the epigenetic silencing of the viral genome (58).

Independent of viral gene expression, IFI16 recognized the HSV-1 genome in infected cell nuclei, re-localized, and co-localized with ASC in the cytoplasm (35, 59). However, HSV-1 specifically targeted IFI16 for rapid proteasomal degradation during post-infection, which was dependent (in certain cell type) on the expression of ICP0, an immediate early protein of HSV-1 (55, 60). In contrast, the levels of expression of NLRP3, AIM2, and ASC were not decreased, Caspase-1 was “trapped” in actin clusters at later time points that most likely blocked the NLRP3/IFI16 inflammasome activity, and inhibited the production of biologically active IL-1β and IL-18 (35). These results suggest that through the host cell response to HSV-1 infection by IFI16 and NLRP3 inflammasomes early during infection, HSV-1 has evolved immune evasion mechanisms to shut down these responses to evade harmful inflammatory responses.

The present report demonstrates that virulent and less-virulent strains of HSV-1 induced different levels of inflammasomes expression along with different levels of Caspase-1 activation and IL-1β production. However, these results do not imply that the activation of specific inflammasomes is directly responsible for the severity of corneal herpetic disease observed with the virulent HSV-1 strains. Nevertheless, the findings that there is an association between the high levels of inflammatory responses, on one hand, and the severity of disease induced by virulent HSV-1 strains, on the other hand, is an important discovery by itself. Our data showed, both *in vitro* and *in vivo*, a clear-cut correlation between high expression of different inflammasomes-triggering proteins (i.e., NLRP3, NLRP12, and IFI16) induced by the most virulent strains, followed by caspase-1 activation (i.e., cleaved caspase-1 as detected in western blot and FLICA staining) and by production of the active form of IL-1β. We are currently investigating direct links between the activation of specific inflammasomes and the severity of corneal disease triggered by virulent strains of HSV-1. In these experiments we are: (i) comparing inflammation and severity of ocular disease induced by various virulent and less-virulent strains of HSV-1 in wild type mice vs. NLRP3, NLRP6, NLRP12, IFI16 and AIM2 deficient mice (28, 46, 61–64); (ii) using co-immunoprecipitation experiment to establish the association/oligomerization of the ASC adaptor with the NRLP-3, NLRP6, NLRP12, IFI16, and AIM2 inflammasomes (26, 65–67); and (iii) performing siRNA knockdown of specific inflammasomes, both *in vitro* and *in vivo* (68–71). The results from these studies will be the subject of a future report.

Activated inflammatory cell infiltrates in HSV-1-infected corneas, with or without herpes stromal keratitis, have been demonstrated using a single strain of HSV-1 (21, 22, 72–78). To the best of our knowledge, no report had compared side-by-side virulent and less-virulent strains of HSV-1 in recruiting inflammatory cells into the cornea and how that affect corneal herpetic disease. The present study extends those reports by showing that the HSV-1 virulence promotes the recruitment of CD45^high^Ly6C^+^Ly6G^−^F4/80^+^CD11b^+^CD11c^−^ macrophages and CD45^high^CD11b^+^F4/80^−^Ly6G^hi^Ly6C^med^ neutrophils infiltrates to infected cornea as early as 2-day post-infection and that this was associated with severe corneal disease. The larger size of CD45^high^Ly6C^+^Ly6G^−^F4/80^+^CD11b^+^CD11c^−^ macrophages and CD45^high^CD11b^+^F4/80^−^Ly6G^hi^ Ly6C^med^ neutrophil infiltrates induced by virulent strains (McKrae, 17, KOS79) correlated with the severity of corneal disease. Previous studies suggest that PMNs play an essential role in the development of corneal infiltrates in stromal herpes virus (HSV) keratitis (37, 73, 79, 80). Corneal infiltration has rarely been observed in herpes-infected animals treated with a PMN-specific mAb (RB6-8C5) or with chemotherapy to reduce the numbers of circulating PMNs (37, 73, 79, 80). In agreement with an early report by Rouse (10), we found the neutrophil infiltration in the cornea, which lasts 2-3 days before they disappear, to be an earliest sign of disease. However, a massive secondary neutrophil infiltration occurs, around day 8 of post-infection and this was associated with a clinical inflammatory HSK. Moreover, we demonstrated that HSV-1 virulence promotes Caspase-1 activation in innate immune cells (inflammatory monocytes and neutrophils) infiltrating the corneas following infection with virulent strains of HSV-1. At day 8 post-infection, we also observed some significant differences in the number of CD3^+^CD4^+^ T cells that infiltrates the cornea of B6 mice infected with virulent and less-virulent strains. This result is in agreement with previous reports showing the involvement of CD4^+^ T cells in the immunopathology of cornea herpetic disease [reviewed in Rajasagi and Rouse (81)].

Viral genetic differences likely impact the inflammasome activation that lead to a varied severity of disease outcomes seen in infected symptomatic (SYMP) and asymptomatic (ASYMP) individuals, but the contributions of naturally-occurring viral variations to herpetic disease are not yet known. A recent genome-wide investigation showed no significant associations between human genetic variation and HSV-2 virologic severity (82). Yet-to-be-determined different virulent factors produced by virulent strains may lead to the simultaneous activation of different inflammasomes (i.e., NLRP3, NLRP12, and IFI16) and this would lead to overwhelming inflammatory cell infiltrates to the infected cornea that translates into a harmful inflammatory herpetic disease. Using viral genome sequencing, we are currently studying the association of viral genetic loci with high- vs. low-virulence and its association with clinical and sub-clinical corneal inflammation in a mouse model of ocular herpes infection, as recently reported by Bowen et al. (19). Virulence is not an intrinsic property but rather depends on both viral and host factors (19, 20, 83). Under similar conditions and using our B6 mouse model of ocular herpes we confirmed previous reports ranking the strains 17, McKrae, and KOS79 as the most virulent strains while F, RE, KOS and KOS63 as less-virulent strains (84–86). Although the virulent clinical isolate of HSV-1 KOS79 and the less-virulent clinical isolate of HSV-1 KOS63 were genetically distinct, they were isolated from the same individual on separate occasions (85). Because US9 appeared to contribute to KOS79 neurovirulence (85) we are currently investigating the potential activation of inflammasomes by US9 product.

The inflammasome-virulence-gene associations and their impacts on viral fitness, inflammasome activation, inflammatory responses and virulence will be subject of a future report. Finally, it is not excluded that ocular surface microbiome and the wide range of ocular microbial DNA-based “signatures” recently reviewed by St. Leger et al. (87), also affect the level and type of inflammasomes activated following ocular infection with the virulent and less virulent strains of HSV-1. There are several implications of the finding that the level of expression of NLRP3, NLRP12, and IFI16 inflammasomes is increase following infection virulent HSV-1 strains and this was associated with severe corneal inflammatory herpetic disease. In SYMP individuals, the NLRP3, NLRP12, and IFI16 pathways might be induced by yet-to-be-determined virulent clinical isolates of HSV-1 causing clinical corneal inflammatory herpetic disease often seen in this group of patients. Higher MOIs (i.e., 3, 5, and 10 MOIs) produced fast and significant death in hTCEpi cells regardless of the strain used. Although the low MOI of 1 did not result in a productive infection in all hTCEpi cells; it led to 63–88% of hTCEpi cells that were HSV-1 positive, depends on the strain used. Since the THP-1 cells appeared to be more resistant to HSV-1 (14), we used a high MOI of 10 (instead of MOI of 1) to infect these refractory cells. Although the kinetics of inflammasomes expression appeared to be different in THP-1 and hTCEpi cells infected with virulent vs. less-virulent strains of HSV-1, a similar trend of inflammasomes expression and Caspase-1 activation was detected THP-1 and hTCEpi cells. Similar to innate immune cells, the THP-1 cell line expresses highs level of inflammasomes proteins (14), but the underlying mechanisms to why the kinetics of some inflammasome expression, particularly the NLRP6, is different between hTCEpi and THP-1 cells remain to be fully elucidated. Moreover, we are also determining whether these inflammasome pathways will be associated with the severity of eye disease and the neuroinvasiveness, comparing wild type B6 mice to NLRP3, NLRP6, NLRP12, AIM2, and IFI16 inflammasomes deficient mice (KO mice). Over the last few years, we have focused on determining how factors in the host's immune system, particularly T cells, affect SYMP and ASYMP individuals with herpes infections (9, 88, 89). Besides potential differences in the immune system between SYMP and ASYMP individuals, it is likely that clinical isolates from SYMP but not from ASYMP have a specific genotypic profile with potential genes (or factors) of virulence.

In conclusion, the experiments described in this study suggest cornea infection with virulent strains of HSV-1 induced simultaneous early expression of the NLRP3, NLRP12, and IFI16 inflammasomes, followed by a Caspase-1 activation that triggered recruitment of neutrophils and inflammatory macrophages into the inflamed cornea. This exaggerated inflammatory response, following corneal infection with virulent HSV-1 strains, in turn lead to clinical corneal inflammatory herpetic disease, irrespective of the level of virus replication in the cornea. An important avenue of this study will be to determine the viral virulent factors that lead to simultaneous sensing and activation of specific inflammasomes that may cause corneal inflammatory herpetic disease. Identification of these virulent viral factors and associated specific activated inflammatory pathways would open new avenues to innovative immunotherapies to inhibit corneal inflammation and alleviate blinding corneal herpetic disease, while at the same time limiting collateral tissue damage.

## Data Availability

The raw data supporting the conclusions of this manuscript will be made available by the authors, without undue reservation, to any qualified researcher.

## Ethics Statement

Female C57BL/6 (B6) wild type (WT) mice (6–8 weeks old) were purchased from The Jackson Laboratory (Bar Harbor, ME). Both male and female mice at 6-8 weeks were used for this study. Animal studies conformed to the Guide for the Care and Use of Laboratory Animals as published by the US National Institute of Health. Animal studies were conducted with the approval of the Institutional Care and Use Committee of University of California-Irvine (Irvine, CA) and conformed to the Guide for the Care and Use of Laboratory Animals published by the US National Institute of Health (IACUC protocol #2002-2372).

## Author Contributions

P-GC, ND, SR, RS, EP, and LB: conceived and designed the experiments. P-GC, ND, RS, NA, AN, WW, CY, EC-C, UM, AC, and KE: performed the experiments. P-GC, ND, RS, SP, and LB: analyzed the data. P-GC, ND, SR, SP, RS, and LB: contributed reagents, materials, and analysis tools. P-GC, ND, and LB: wrote the paper.

### Conflict of Interest Statement

The authors declare that the research was conducted in the absence of any commercial or financial relationships that could be construed as a potential conflict of interest.

## Supplementary Material

Supplemental Figure S1 Gating strategies. **(A)** Represents the gating strategy used in Figure 2: CD45 was used to gate leukocytes infiltrating the infected corneas. The neutrophil population was determined by the expression of CD11b^+^ and Ly6G^+^ among the F4/80^−^/Ly6C^med^ cells. We then gated the inflammatory monocytes inside the F4/80^+^ CD11c^−^ Ly6G^−^ population, looking at the Ly6C^high^ / CD11b^+^ cells. **(B)** represents the gating strategy used in Figure 7.

Supplemental Figure S2 Fold change variation overtime in the numbers of neutrophils and inflammatory monocytes infiltrating the cornea. Fold change variation in the size of the neutrophil **(A)** and inflammatory monocyte **(B)** corneal infiltrate between day 2 and day 8 after infection with 2.5 × 10^5^ pfu/eye of HSV-1 laboratory strains (i.e., McKrae, 17, F, RE or KOS) or clinical isolates (i.e., KOS63 or KOS79). *P*-values were determined using ANOVA-multiple-comparison-test.

Supplemental Figure S3 Expression level of NLRP3, NLRP6, NLRP12, IFI16, AIM2, Caspase-1, IL-1β and IL-18 in THP-1 derived macrophages infected with virulent and less-virulent strains of HSV-1. THP-1 (PMA treated) derived macrophages were infected *in vitro* with the HSV-1 laboratory strains McKrae, 17, F, RE, and KOS or with the clinical isolates KOS79 and KOS63 at an MOI of 10. At two, 8 and 24 h post-infection (**A**–left, center and right panels respectively), the cells were harvested and immunoblots of whole cell lysates were performed for the expression of the NLRP3, NLRP6, NLRP12, IFI16 and AIM2 inflammasomes, along with the expression of pro- /cleaved - Caspase-1, pro- /cleaved - IL-1β and IL-18. Corresponding β-actin was used as a control **(B)** Graphs show the kinetics of fold changes in expression of NLRP3, NLRP6, NLRP12, IFI16, AIM2, Caspase-1, IL-1β, and IL-18 at two, 8 and 24 h post-infection with virulent/less-virulent strains of HSV-1 compared to the control (mock) and normalized to β-actin. **(C)** IL-1β was also measured in culture supernatants by ELISA at two, 4, 8, 24, and 48 h post-infection. Results are representative of three experiments.
