# Supplementary figures and images for: NLRP3, NLRP12, and IFI16 Inflammasomes Induction and Caspase-1 Activation Triggered by Virulent HSV-1 Strains Are Associated With Severe Corneal Inflammatory Herpetic Disease
Source: Front Immunol. 2019 Jul 16;10:1631. doi: 10.3389/fimmu.2019.01631 (PMC6644090; doi:10.3389/fimmu.2019.01631)

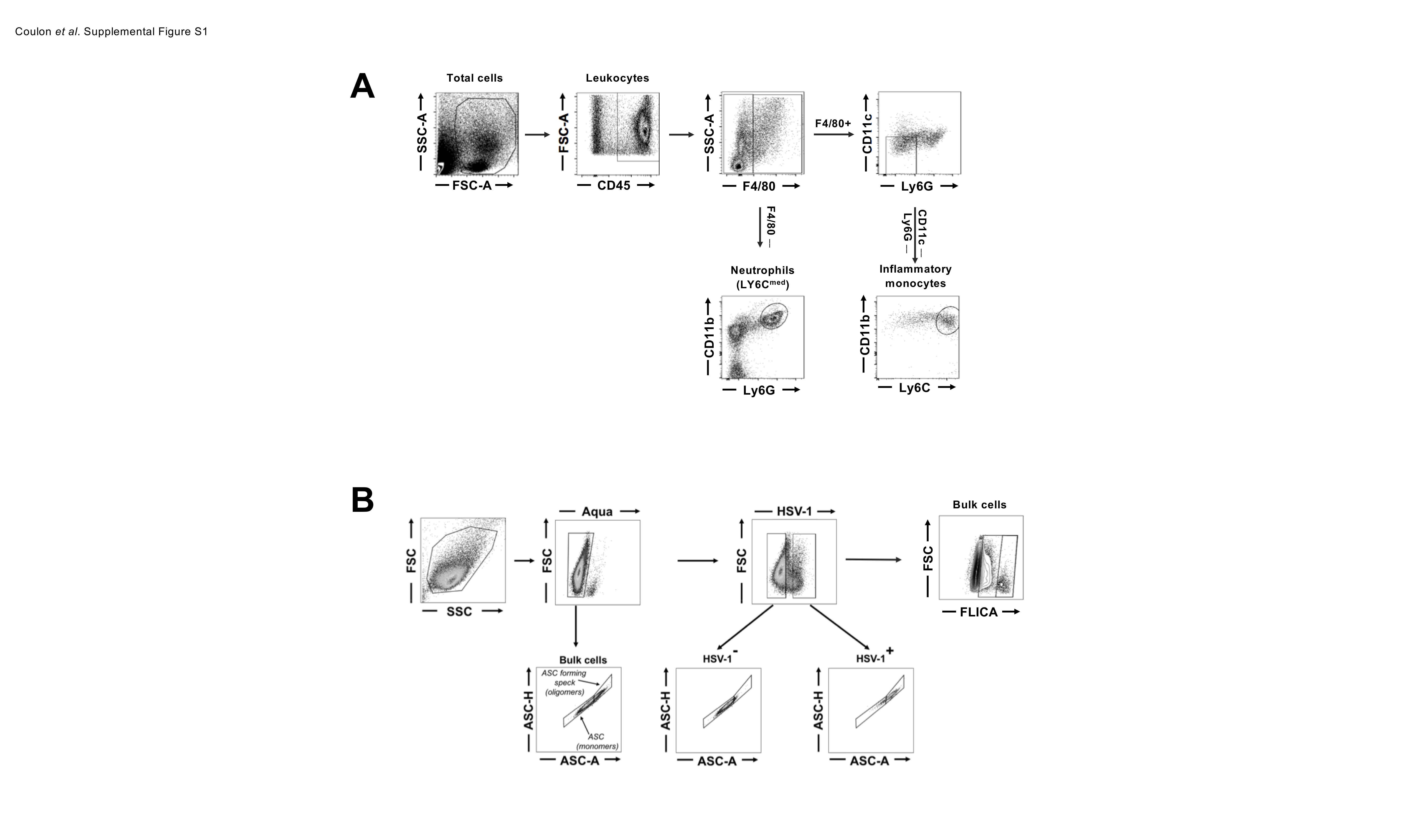

Supplement: Supplemental Figure S1 — Gating strategies. (A) Represents the gating strategy used in Figure 2: CD45 was used to gate leukocytes infiltrating the infected corneas. The neutrophil population was determined by the expression of CD11b+ and Ly6G+ among the F4/80−/Ly6Cmed cells. We then gated the inflammatory monocytes inside the F4/80+ CD11c− Ly6G− population, looking at the Ly6Chigh / CD11b+ cells. (B) represents the gating strategy used in Figure 7. [file Image_1.TIFF]

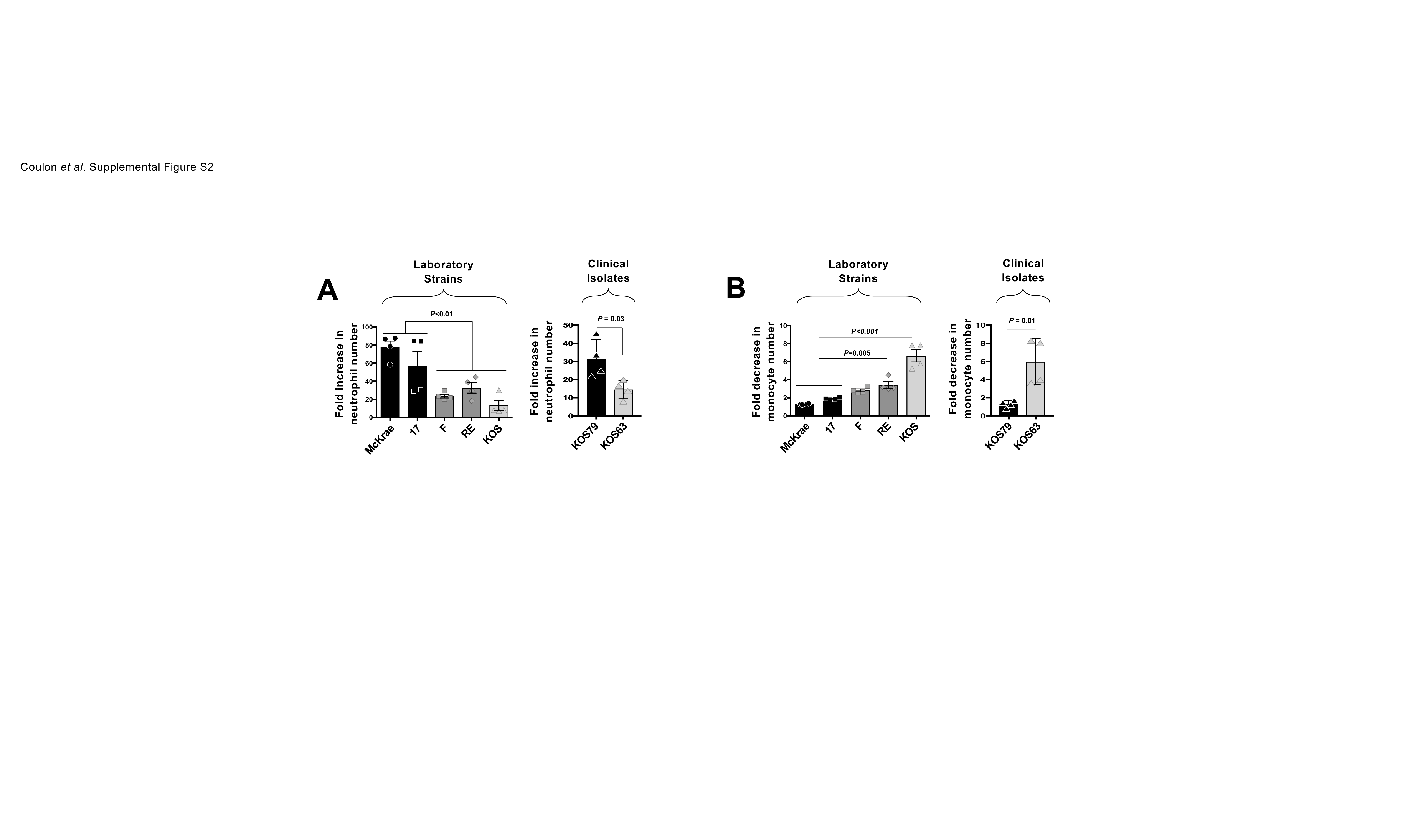

Supplement: Supplemental Figure S2 — Fold change variation overtime in the numbers of neutrophils and inflammatory monocytes infiltrating the cornea. Fold change variation in the size of the neutrophil (A) and inflammatory monocyte (B) corneal infiltrate between day 2 and day 8 after infection with 2.5 × 10^5^ pfu/eye of HSV-1 laboratory strains (i.e., McKrae, 17, F, RE or KOS) or clinical isolates (i.e., KOS63 or KOS79). P-values were determined using ANOVA-multiple-comparison-test. [file Image_2.TIFF]

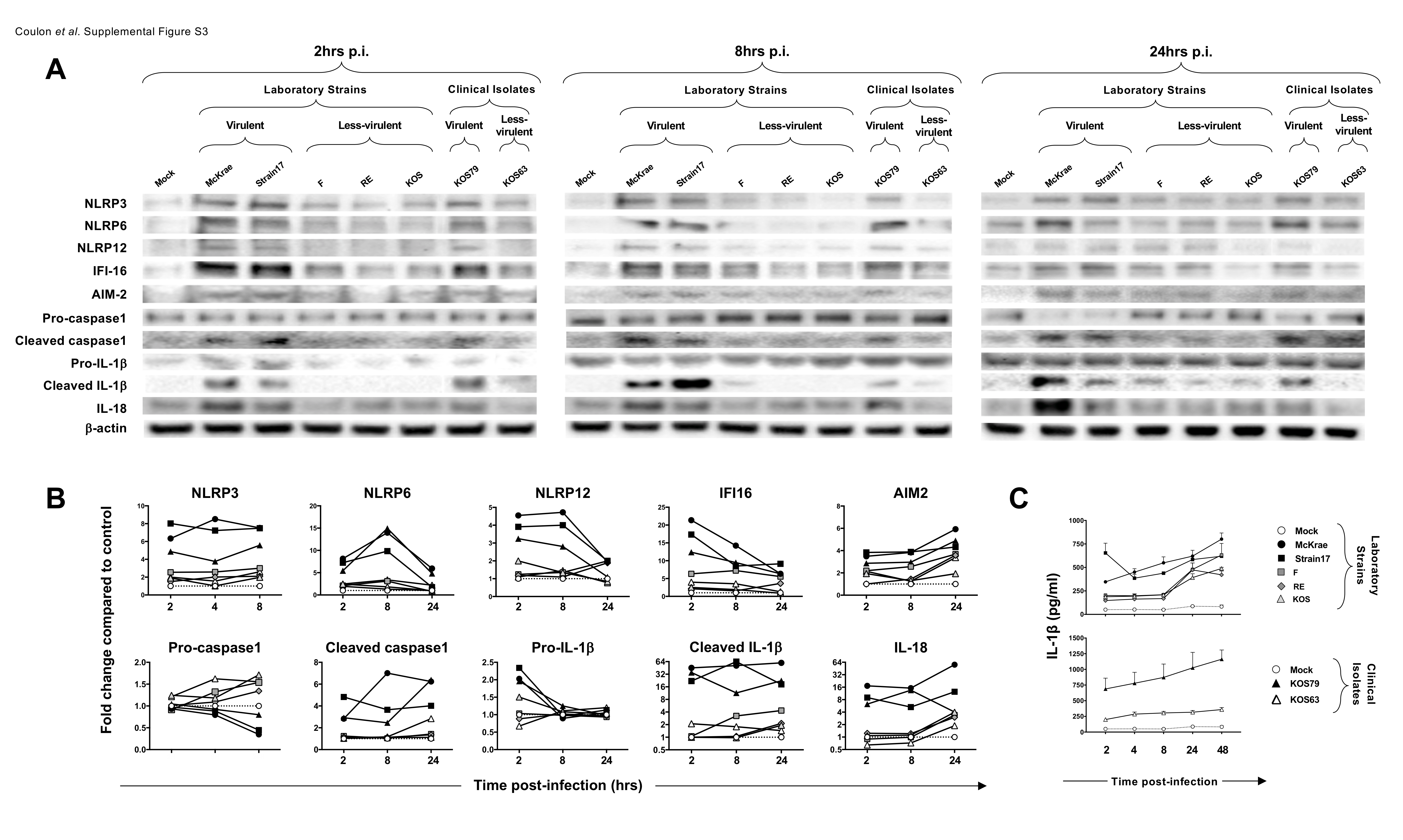

Supplement: Supplemental Figure S3 — Expression level of NLRP3, NLRP6, NLRP12, IFI16, AIM2, Caspase-1, IL-1β and IL-18 in THP-1 derived macrophages infected with virulent and less-virulent strains of HSV-1. THP-1 (PMA treated) derived macrophages were infected in vitro with the HSV-1 laboratory strains McKrae, 17, F, RE, and KOS or with the clinical isolates KOS79 and KOS63 at an MOI of 10. At two, 8 and 24 h post-infection (A–left, center and right panels respectively), the cells were harvested and immunoblots of whole cell lysates were performed for the expression of the NLRP3, NLRP6, NLRP12, IFI16 and AIM2 inflammasomes, along with the expression of pro- /cleaved - Caspase-1, pro- /cleaved - IL-1β and IL-18. Corresponding β-actin was used as a control (B) Graphs show the kinetics of fold changes in expression of NLRP3, NLRP6, NLRP12, IFI16, AIM2, Caspase-1, IL-1β, and IL-18 at two, 8 and 24 h post-infection with virulent/less-virulent strains of HSV-1 compared to the control (mock) and normalized to β-actin. (C) IL-1β was also measured in culture supernatants by ELISA at two, 4, 8, 24, and 48 h post-infection. Results are representative of three experiments. [file Image_3.TIFF]
